# Glucose as a cause of and treatment for cutaneous necrosis

**DOI:** 10.1590/1677-5449.004818

**Published:** 2018

**Authors:** Marcelo Luiz Brandão, Amina Muhamad Mota Mustafá, Jordana Lopes Costa

**Affiliations:** 1 Pontifícia Universidade Católica de Goiás - PUC Goiás, Goiânia, GO, Brasil.

**Keywords:** wound healing, sclerotherapy, glucose, telangiectasis, skin ulcer, petrolatum

## Abstract

Sclerotherapy remains one of the procedures most frequently performed by Brazilian vascular surgeons. Knowledge of its complications is indispensable to enable us to avoid them. The severe side effects of this method of treatment for telangiectasias of the lower limbs are rare and are often associated with technical errors or the dose injected. Complications are predominantly local, but are sometimes difficult to resolve. We report a case of formation of cutaneous necrosis after chemical sclerotherapy using hypertonic glucose (75%), which healed when treated with a topical preparation containing vaseline and 60% glucose, with satisfactory esthetic results.

## INTRODUCTION

 Chemical sclerotherapy for treatment of telangiectasias of the lower limbs consists of intravascular introduction of a liquid with the aim of provoking injury and subsequent luminal occlusion. 1 ^,^ 2 The ideal result is uniform destruction of the entire endothelium, followed by fibrosis with minimal formation of thrombus. Its relative technical simplicity and reproducibility means that this treatment modality can be used as a treatment option for patients with telangiectasias (CEAP C1) and/or varicose veins (CEAP C2) in the lower limbs. 3 ^,^ 4

 Endothelial damage can be provoked by changing pH and osmolarity (which alter plasmatic membrane surface tension) or by direct lesion of the endothelium. On the basis of these objectives, sclerosant solutions can be grouped into three categories: osmotic agents, detergents, and chemical irritants. 1

 Hypertonic osmotic solutions cause endothelial dehydration and disintegration and denature the cell membrane. They also act to disperse fibrinogen from the tunica intima, depositing fibrin on the vein interior and around the vein wall, provoking its collapse and disappearance. 5 ^-^ 9 Objectively, an inflammatory reaction sets in and gradually progresses to fibrosis. 

 Hypertonic glucose was introduced by Kausch in 1917, is one of the agents most widely used for this purpose in Brazil, 10 and is routinely employed because of its efficacy, low cost, and almost nonexistent side effects, such as necrosis or allergic reactions. However, repeated injections into the same vessel during successive sessions at varying intervals may be needed, 11 which, in theory, increases the risk of complications. 

 Because hypertonic glucose is rarely used in Europe or the United States, there is relatively little literature on it. 11 This is one of the reasons that prompted us to describe a complication that occurred after glucose was employed and then treated using glucose. 

### Objective

 To report a case of cutaneous necrosis after sclerotherapy for telangiectasias in the lower limbs, using hypertonic glucose (75%) and its healing in response to treatment using topical 60% glucose. 

## CASE DESCRIPTION

 A 49-year-old female patient (Fitzpatrick III phototype) sought medical care in November 2016 complaining of varicose veins in the lower limbs, which at the time were asymptomatic. She stated that she had no comorbidities or allergies. She was taking the following medications: 0.100 mg levonorgestrel and 0.020 mg ethinylestradiol. A physical examination only found a moderate quantity of telangiectasias (CEAP C1), predominantly of the arborizing type. An arterial examination was normal. 

**Figure 1:**
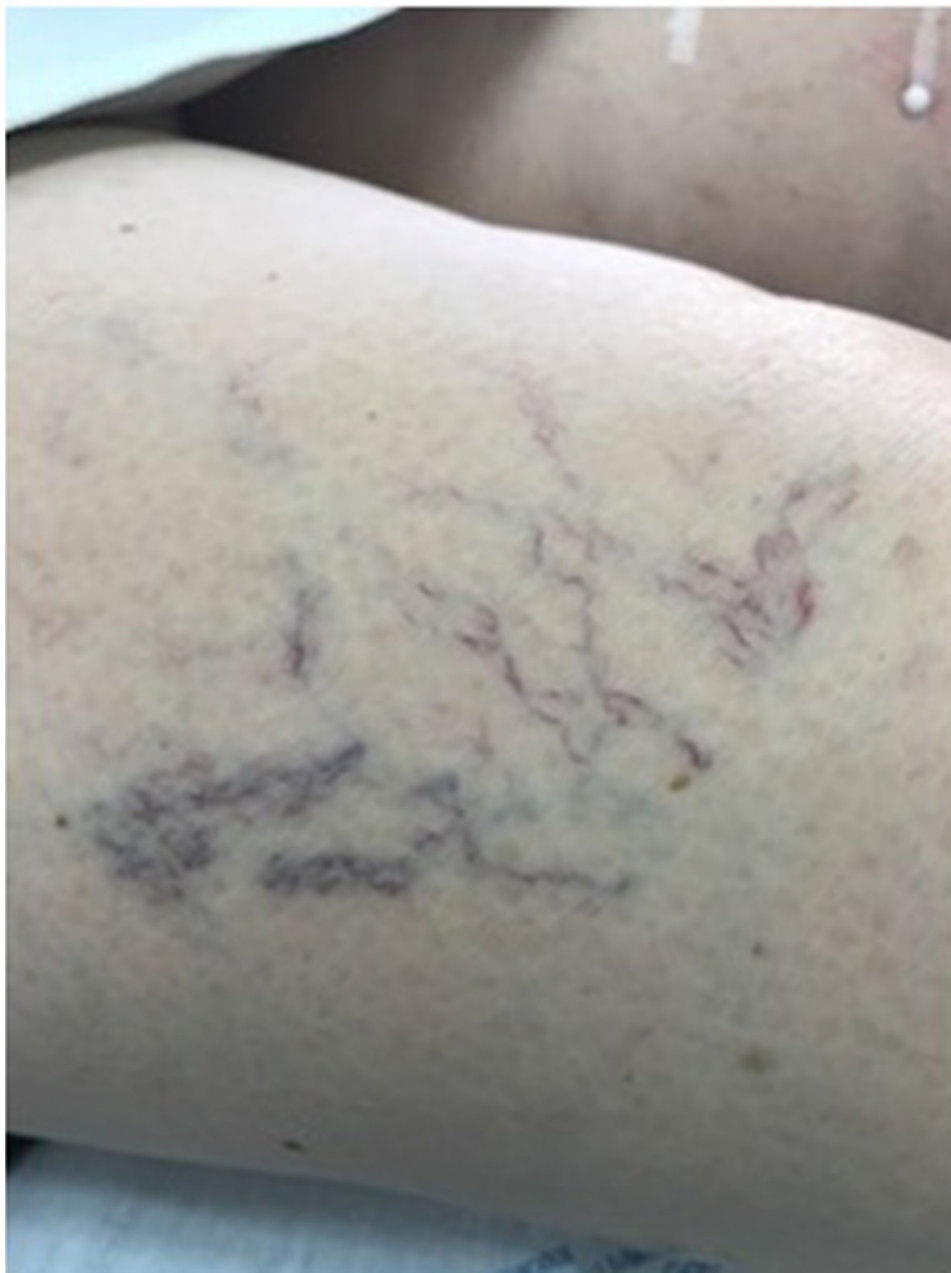
Telangiectasias (arborizing type) and venulectasias in the lateral region of the right thigh (CEAP 1).

**Figure 2:**
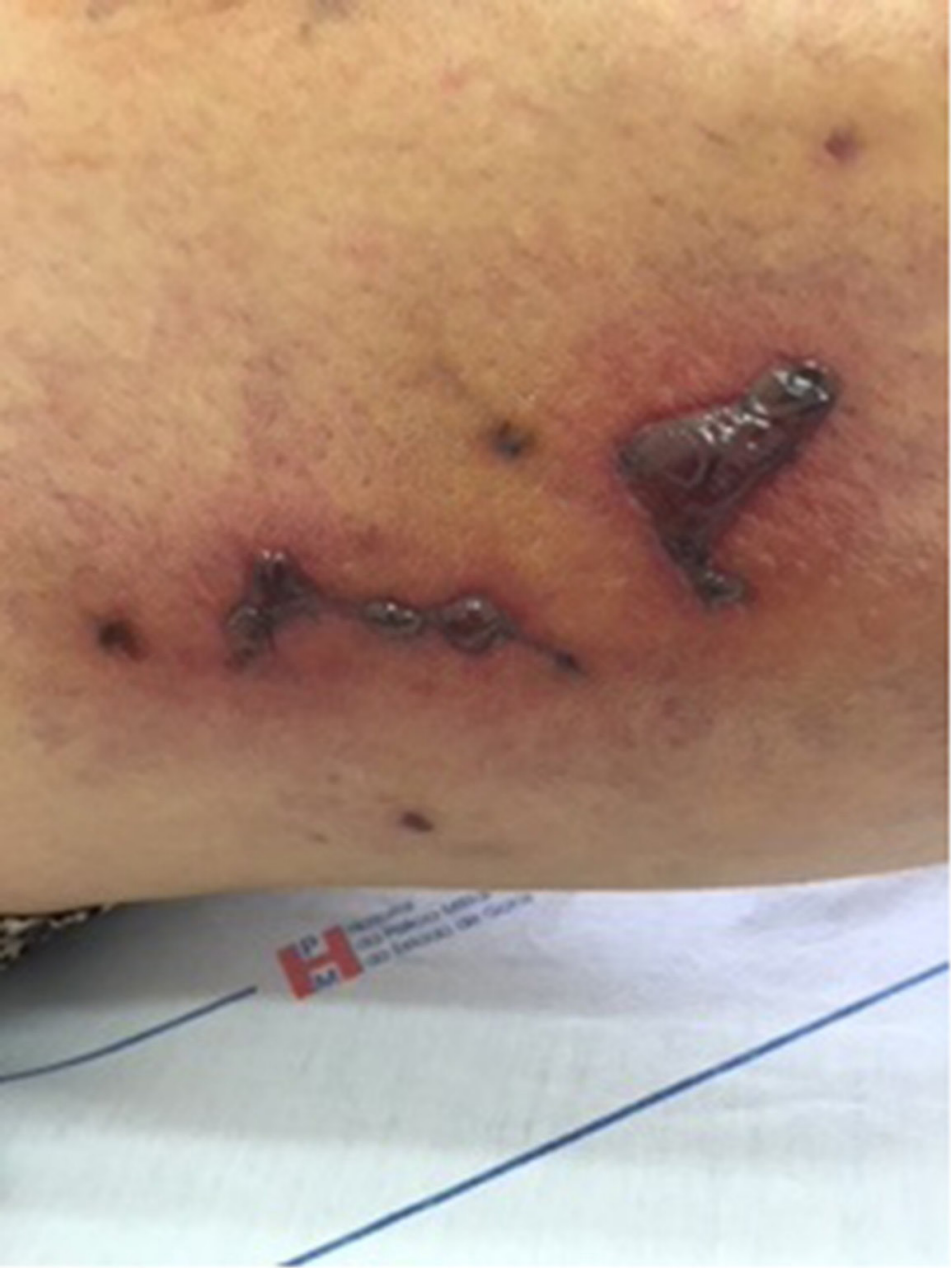
Erythema and blisters 7 days after the injection of 75% glucose for esthetic treatment of CEAP 1 telangiectasias.

 The purpose of treatment was essentially esthetic. In March 2017, the first sclerotherapy session was conducted with 75% glucose (at a temperature of 17 °C, achieved in advance) using a 0.40 x 13 mm (27G x ½”) needle and a 3 mL syringe (Total volume = 2 mL). Around 10 minutes after the injection into the lateral region of the right thigh, where the concentration of telangiectasias was greatest ( Figure 1 ), an ochre-colored stain was observed. It progressed with formation of blisters and erythema ( Figure 2 ), which were observed on the seventh day after sclerotherapy. 

 The patient also exhibited pain, edema (+ / +4), and clubbing (++ / +4) of the ipsilateral calf, all with simultaneous onset. Superficial thrombi were drained (maintaining the blisters intact) and a color Doppler ultrasonography examination was conducted because of a suspicion of deep venous thrombosis, which was ruled out. The patient had been instructed to wear elastic stockings (20-30 mmHg compression) after the initial sclerotherapy, but was then proscribed from wearing them on the seventh day after sclerotherapy, when edema and skin lesions were observed. 

**Figure 3:**
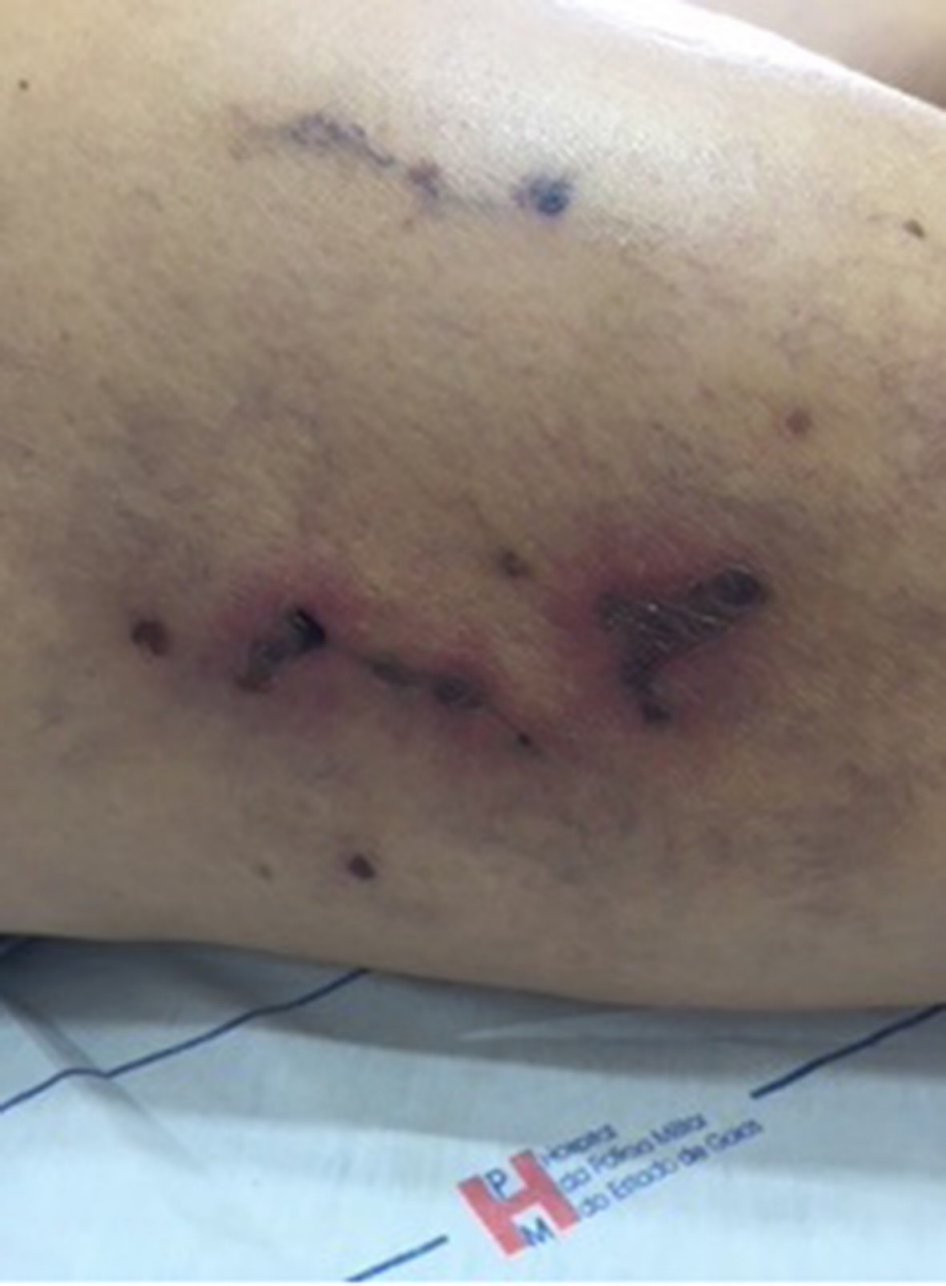
Formation of scabs on the 14th day after sclerotherapy with hypertonic glucose (75%).

**Figure 4:**
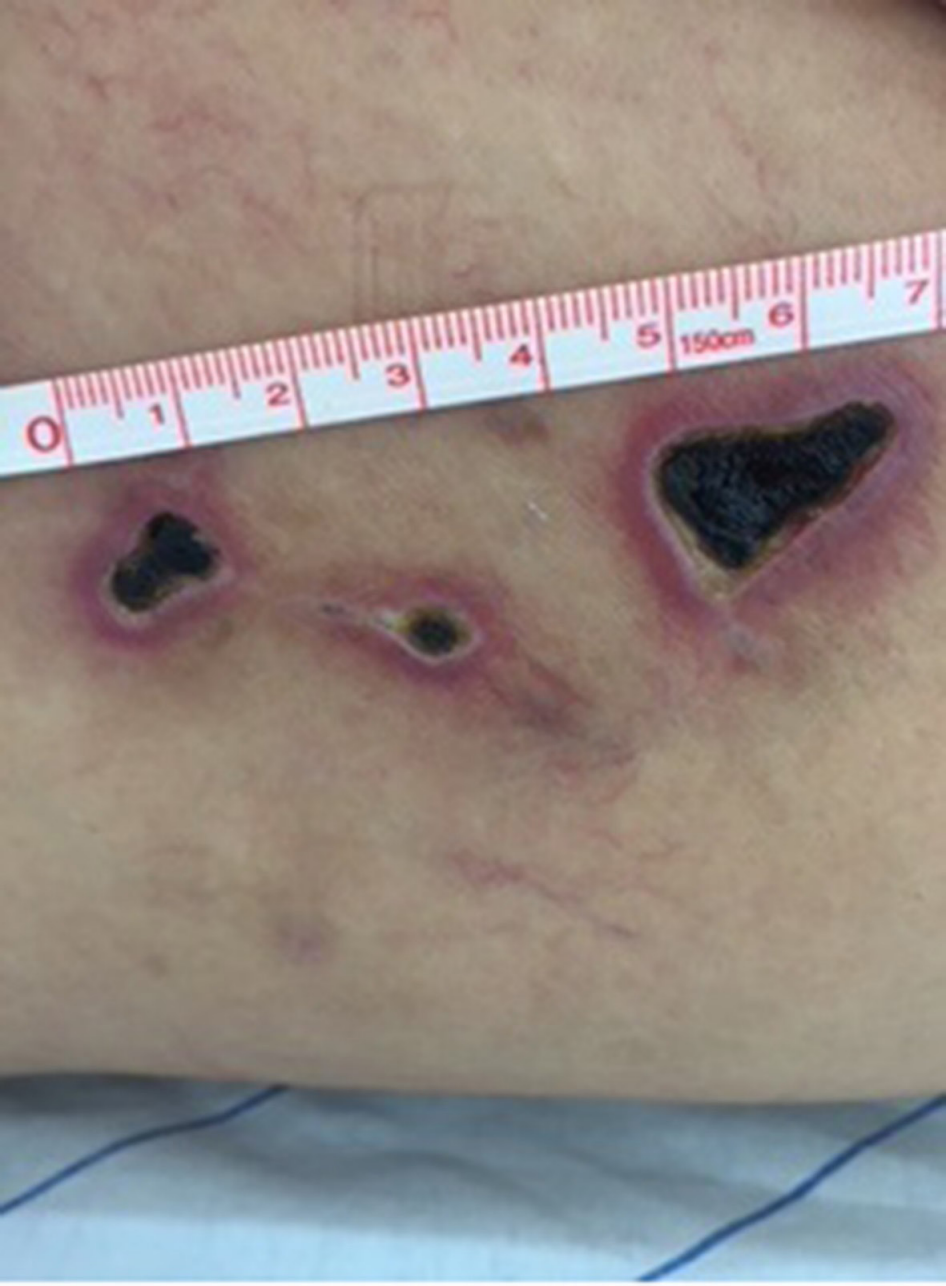
Development of scabs into necrosis by 33rd day after esthetic sclerotherapy for telangiectasias of the lower limbs using hypertonic glucose (75%).

**Figure 5:**
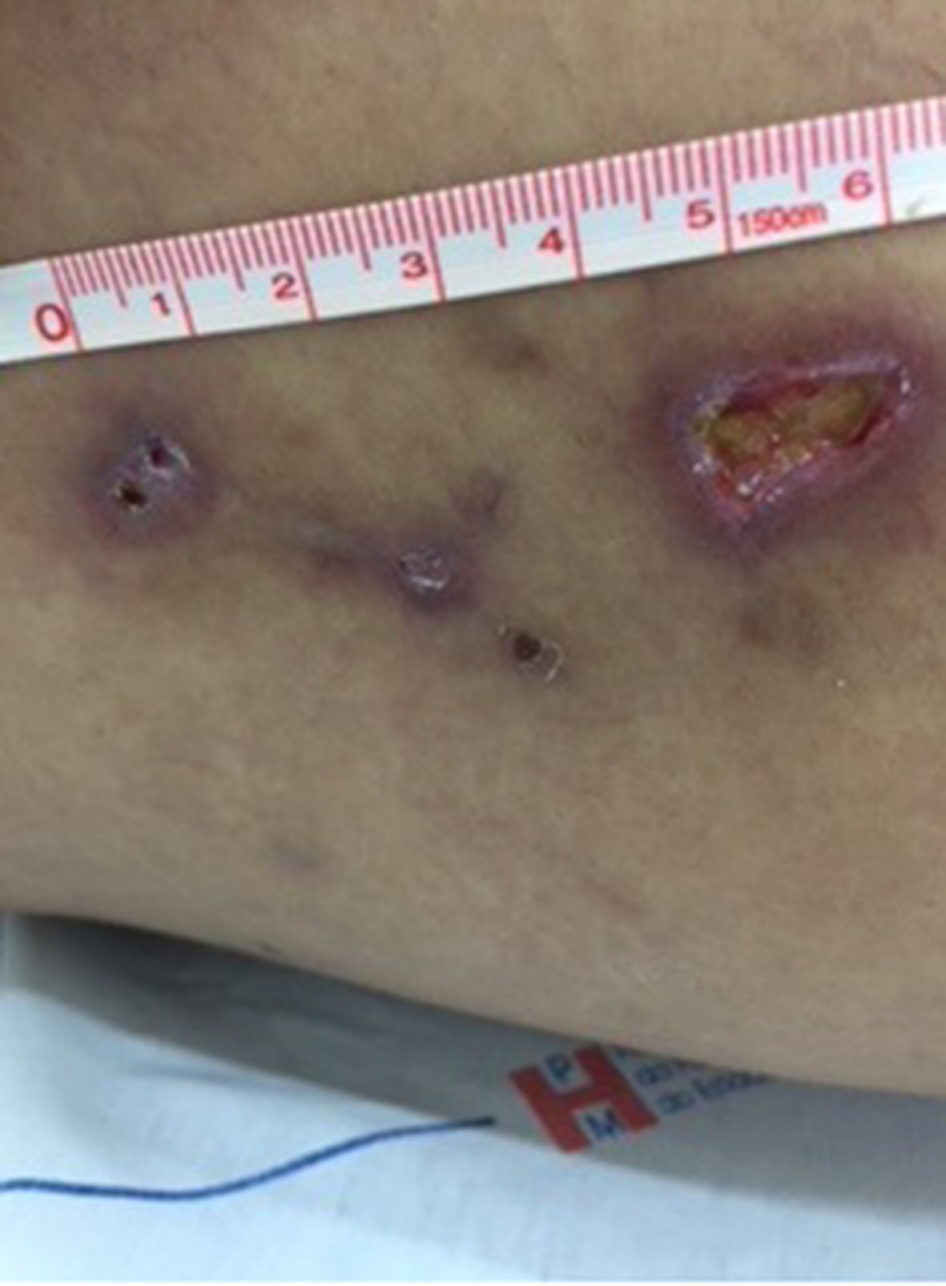
After mechanical debridement of cutaneous necrosis caused by esthetic sclerotherapy for telangiectasias of the lower limbs using hypertonic glucose (75%).

**Figure 6:**
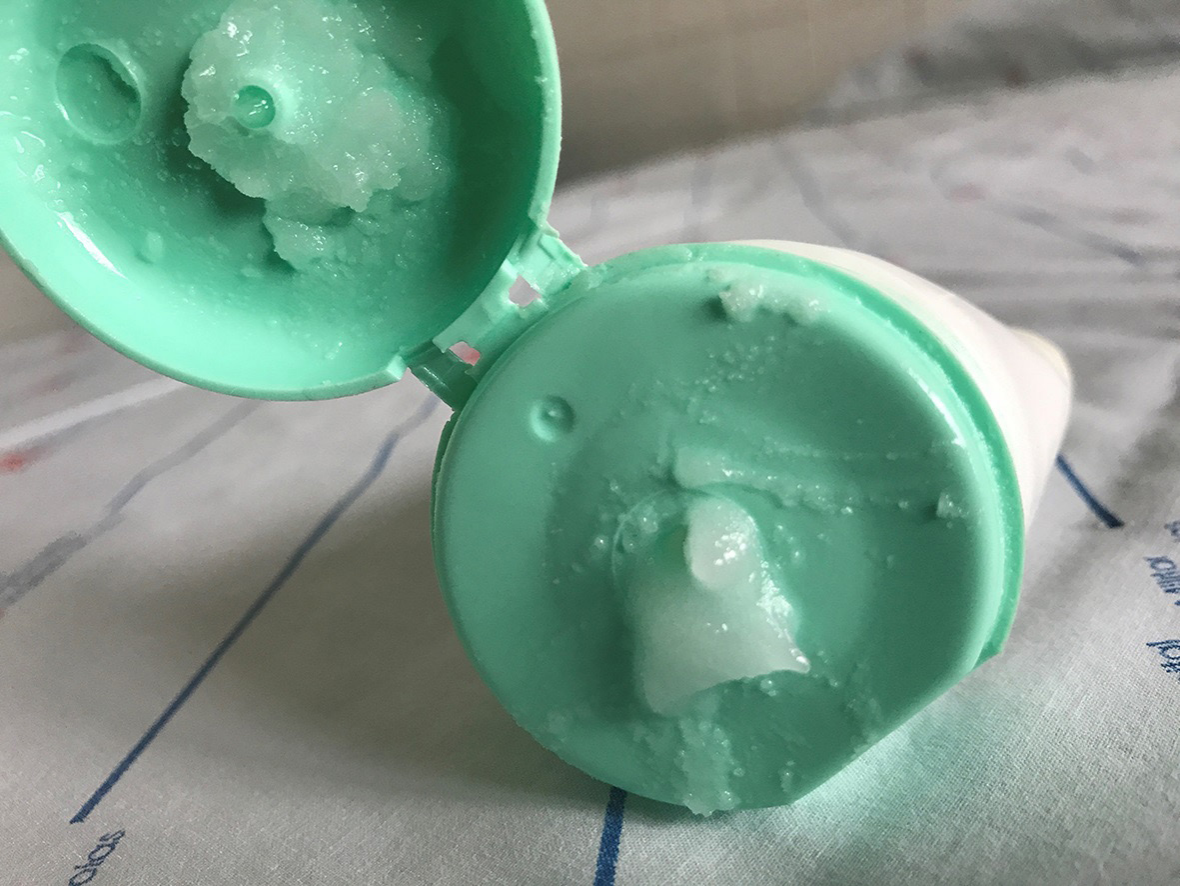
Topical preparation comprising 60% glucose + 40% vaseline.

 On the 14th day after sclerotherapy, the pain, erythema, and edema had improved, but scabs ( Figure 3 ) had appeared where the blisters had been. The patient was instructed to apply dressings daily using oil containing essential fatty acids (EFAs). Formation of necrosis ( Figure 4 ) prompted mechanical debridement, on the 42nd day after sclerotherapy ( Figure 5 ), and the EFAs were withdrawn and daily topical administration of a preparation containing 60% glucose and 40% vaseline 12 was initiated ( Figure 6 ). 

**Figure 7:**
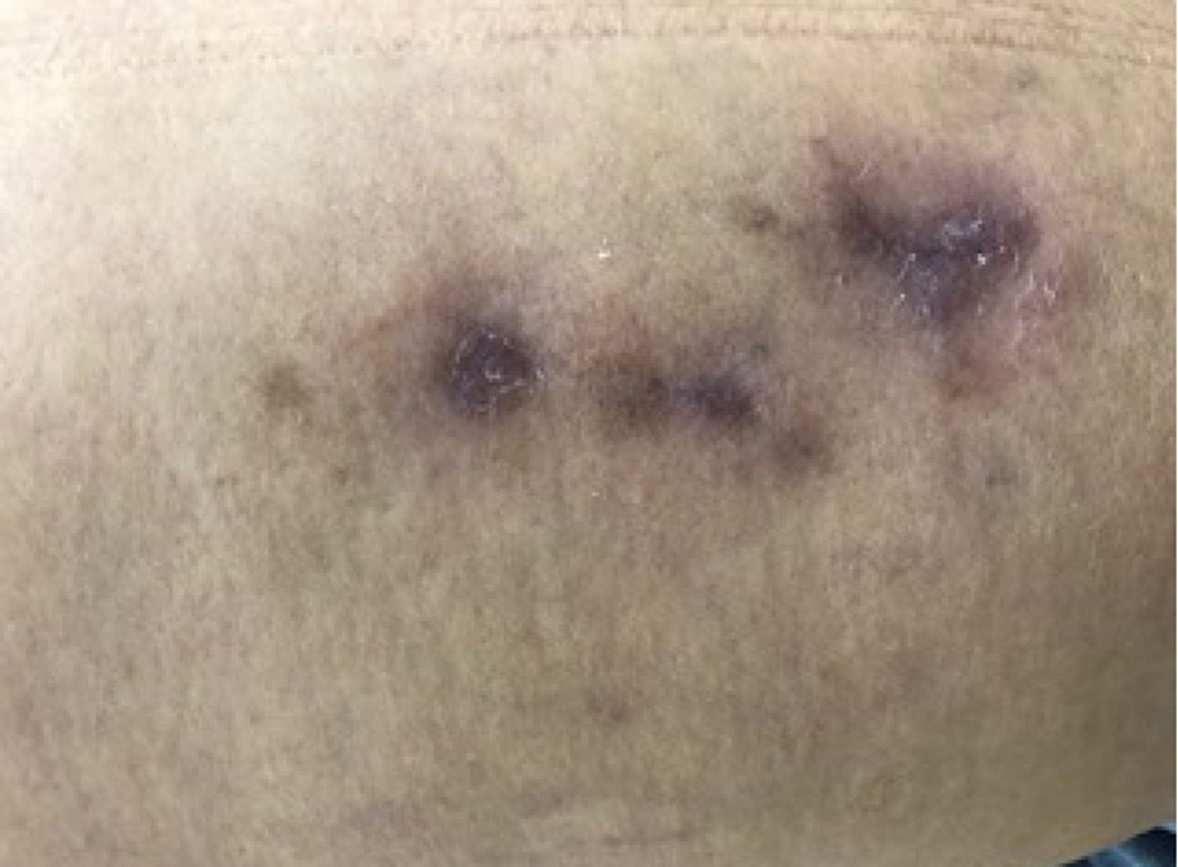
Hyperpigmentation after healing of cutaneous ulcers caused by esthetic sclerotherapy for telangiectasias of the lower limbs using hypertonic glucose (75%) and treated with topical preparation containing 60% glucose.

 A second sclerotherapy session with 75% glucose was conducted on the 49th day after sclerotherapy, when the patient still had an ulcer measuring 2.00 x 1.00 cm, and injections in proximity to this area were avoided. The ulcer had healed by the 88th day after sclerotherapy, but hyperpigmentation remained ( Figure 7 ) and the patient was prescribed hydroquinone, retinoic acid, and hydrocortisone. 

**Figure 8:**
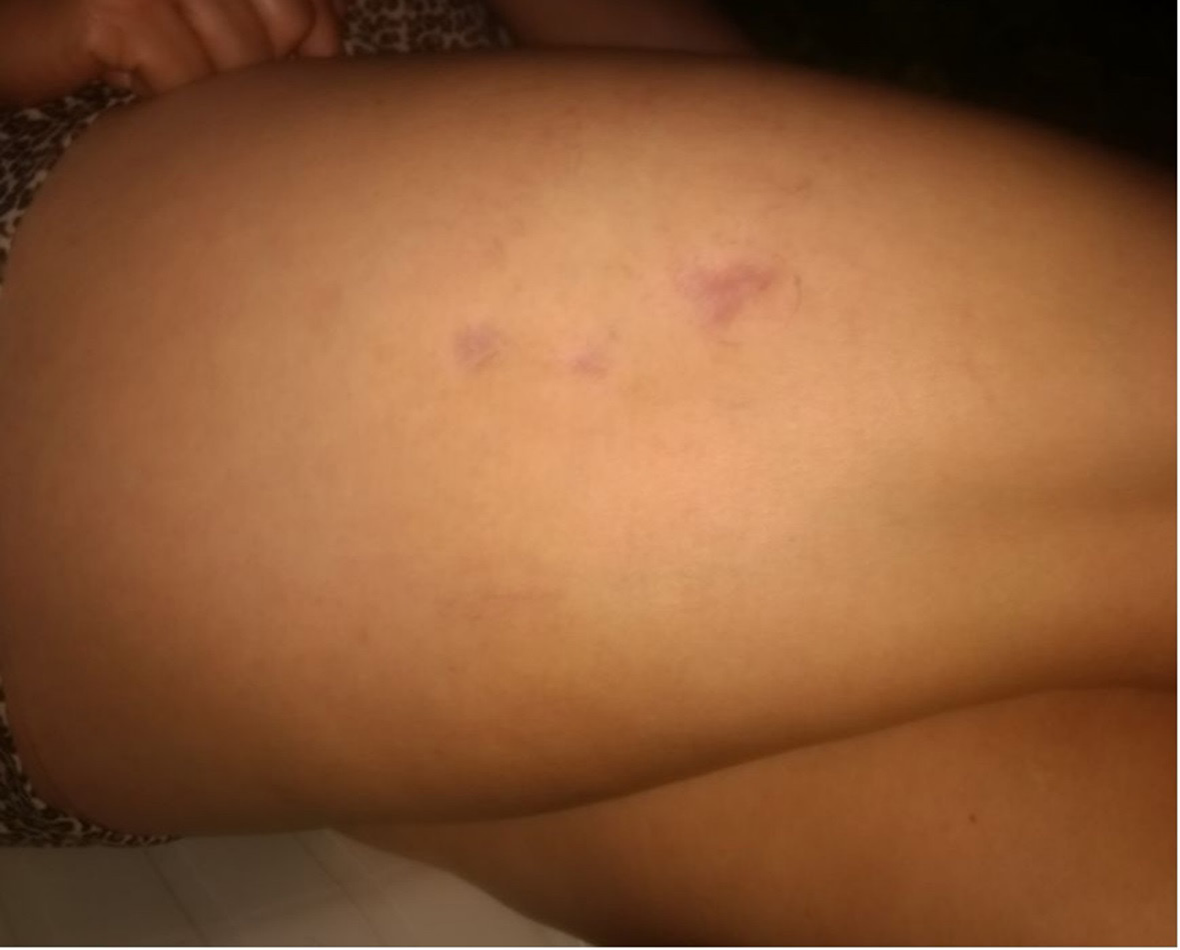
Residual hyperpigmentation after cutaneous necrosis caused by esthetic sclerotherapy for telangiectasias of the lower limbs using hypertonic glucose (75%).

 After using the depigmentation agent for 6 months, the patient exhibited a discrete reduction in pigmentation. At the same time, another sclerotherapy session was conducted with 75% glucose, with no intercurrent conditions. After 1 year, the pigmentation in the scarred area had lightened moderately ( Figure 8 ). The result with relation to the telangiectasias was relatively satisfactory, provoking disappearance of the majority of them. 

## DISCUSSION

 Severe side effects of sclerotherapy are rare and are generally associated with a technical error or with the dosage injected. The complications are predominantly local, such as hyperpigmentation, matting and, more rarely, tissue necrosis and ulceration, 1 which are painful and slow to heal. Sometimes they can be difficult to resolve and have a considerable psychological impact, since in almost all cases the objective of sclerotherapy is esthetic, whereas telangiectasias are unlikely to cause symptoms. 

 The risk of undesirable reactions is directly related to the type of sclerosing agent employed; i.e., the greater its capacity to injure the endothelium, the greater its potential to cause complications. In this respect, hypertonic glucose has proven totally safe. 11 Nevertheless, the possibility of ulcer formation after injection of osmotic agents has been described. 13

 Figueiredo and Figueiredo 10 used a questionnaire to analyze the sclerotherapy techniques and conduct employed by Brazilian angiologists and vascular surgeons. They reported that 5.60% of interviewees had observed ulcers caused by conventional chemical sclerotherapy. However, they did not state which products were used. 

 Cutaneous necrosis can be caused by injection of any sclerosing agent, even under ideal technical conditions and does not necessarily imply a medical error. The exact mechanism of formation is not fully understood. Causes that have been suggested include: (1) leakage of the solution into the perivascular space; (2) injection into arteriole supplying the skin; (3) vasospasm reaction; (4) migration of the sclerosing agent into the arterial bed (arteriovenous anastomoses); (5) occlusion of arteriovenous shunts; or (6) excessive cutaneous pressure produced by incorrect external compression technique. 1

 Miyake 14 associates development of cutaneous necrosis with veno-capillary reflux of the sclerosing solution injected (under excessive pressure), producing vasoconstriction and obstruction of regional microarterioles. If so, necrosis would not therefore be the result of inadvertent intradermal or subcutaneous injection. It would, in this case, be an ischemic phenomenon, which is also confirmed by other authors. 15 ^-^ 17 This hypothesis is based on three pillars 17 :

1 – Presence of cutaneous necrosis even when there is no leakage of the solution;
2 – The cutaneous necrosis exhibits similar behavior to ischemic ulcers caused by arterial occlusion, both in terms of pain and of clinical course;
3 – The more powerful the sclerosing agent, the greater the likelihood of provoking ulceration.

Experimental studies demonstrate that cutaneous necrosis has a direct relationship with injection pressure and an inverse relationship with the diameter of the vessel, i.e., the larger the pressure and the smaller the vessel the more likely it is to occur. According to the Poiseuille Law, pressure reduces in proportion to increased viscosity. Thus, the risk of cutaneous necrosis is lower when high-viscosity sclerosants are used. Osmotic sclerosants are therefore more advantageous than detergents (which are less viscous). 1 ^,^ 17

 However, Munavali and Weiss 18 have suggested that the most common cause of necrosis would be leakage of sclerosing agents into the perivascular territory. Such leakage would cause more traumatic than ischemic ulcers, which would be most frequent when using ethanolamine, followed by polidocanol, and then chromated glycerin, and would be least common with glucose at the varying different hypertonic concentrations it is used at, so it is considered one of the safest agents in terms of adverse reactions, both local and systemic. 1 ^,^ 2 ^,^ 8 ^,^ 9 If 75% glucose leaked, it would cause minor, superficial skin necrosis (1 to 2 mm) that should heal in 1 to 2 weeks. 1 ^,^ 17

 Nevertheless, it is worth mentioning that, as observed in practice, even with no leakage, using the solution habitually employed, and without exerting excessive pressure, there is still a possibility that ulcers will develop, even if the risk of cutaneous necrosis is lower than when sclerosants with greater viscosity (than hypertonic glucose) are used. One possible explanation is a vasospasm reaction (venoarteriolar reflex), causing ischemia. If this condition is suspected, which is not always simple, an attempt can be made to puncture the vein again and flush it with a saline and lidocaine solution, which has a powerful vasodilator effect. 

 We therefore conjecture that the skin necrosis in the case described here was possibly caused by a vasospasm reaction, especially in view of the volume injected to the region (close to 2.00 mL), since neither high pressure injection nor agent leakage occurred. We also consider it to be improbable that any of the following occurred: (1) injection into a dermal arteriole (due to the blue type of telangiectasias); (2) occlusion of arteriovenous shunts; or (3) excessive pressure caused by the elastic stockings (20-30 mmHg) worn during the first 7 days after sclerotherapy. Nevertheless, it is important to point out that migration of the sclerosing agent into the capillary or arteriolar beds cannot be entirely ignored, since assessment of the pressure exerted was subjective. Furthermore, reflux may have occurred as a result of occlusion of the vessels treated (because of the quantity injected). 

 When superficial ulcers occur, they can be treated with a range of products employed to stimulate tissue regeneration, such as creams containing vitamins A or D, aloe vera, zinc oxide, and others. Deeper ulcers normally involve a greater volume of necrotic tissue that requires mechanical and/or autolytic debridement (fibrinolysin, collagenase, calcium alginate and sodium, papain, etc.). 

 To date, there is no consensus on the ideal dressing for treatment of ulcers of vascular origin, particularly not for those caused by sclerotherapy. Motivated by the excellent results reported by Franceschi et al., 12 who applied a pharmaceutical preparation containing glucose and vaseline for topical treatment of chronic lower limb ulcers of varying etiologies (trauma, ischemia, venous hypertension, etc.), and in view of the large size of the cutaneous necrosis and the final depth of the ulcer after mechanical debridement, we chose to employ the same formula, without using any type of systemic drug, not even antibiotics. The concentration of glucose in this combination is 60%, whereas there is almost no glucose in white and brown sugar, which contain 99.8% and 95% sucrose respectively. 12 Furthermore, the vaseline and glucose mixture is not allergenic and is inexpensive. 

 The anti-infectious efficacy and acceleration of wound healing provoked by sugar in ulcer treatment are already known. 19 ^-^ 21 In the case reported here, healing took 46 days. However, in contrast with what was described by Franceschi et al., 12 who changed dressings every 6 or 7 days, we chose to substitute them every 24 hours strictly because of the difficulty of isolating the thigh when bathing, which would have made the area damp. Despite the moderate attenuation of post-healing hyperpigmentation, we continued to use the hydroquinone, retinoic acid, and hydrocortisone, expecting that hyperpigmentation would continue to fade. 

 In conclusion, despite the complication described here, which may have been the result of a preventable technical failure, hypertonic glucose still seems to us to be the safest sclerosing agent in terms of undesirable effects of sclerotherapy. While it may seem contradictory, topical glucose proved to be effective for healing the ulcer provoked by glucose injection and is inexpensive and easy to administer. 
