# Supplementary material for: Glucose as a cause of and treatment for cutaneous necrosis
Source: J Vasc Bras. 2018 Oct-Dec;17(4):341–7. doi: 10.1590/1677-5449.004818 (PMC6375268; doi:10.1590/1677-5449.004818)
Supplement: Translation

# Translation: Glicose como causa e tratamento de necrose cutânea

**Authors:** Marcelo Luiz Brandão, Amina Muhamad Mota Mustafá, Jordana Lopes Costa

**Affiliations:** 1 Pontifícia Universidade Católica de Goiás – PUC Goiás, Goiânia, GO, Brasil.

**Keywords:** cicatrização, escleroterapia, glicose, telangiectasia, úlcera cutânea, vaselina

## Abstract

A escleroterapia continua sendo um dos procedimentos mais executados pelos cirurgiões vasculares brasileiros. O conhecimento das suas complicações é imprescindível para que possamos evitá-las. Os efeitos colaterais graves desse método de tratamento para as telangiectasias dos membros inferiores são raros e frequentemente associados a um erro técnico ou à dosagem injetada. São predominantemente locais, apresentando-se, algumas vezes, como uma situação de difícil resolução. Relatamos um caso de formação de necrose cutânea após escleroterapia química com glicose hipertônica (75%) e sua cicatrização utilizando preparação tópica contendo vaselina e glicose 60%, cujo resultado estético foi satisfatório.

## INTRODUÇÃO

 A escleroterapia química para tratamento de telangiectasias nos membros inferiores consiste em introduzir um agente líquido intravascular com o propósito de provocar lesão e subsequente oclusão luminal 1 ^,^ 2 . Objetiva-se, idealmente, a destruição uniforme de todo o endotélio e a ulterior fibrose, com mínima formação de trombo. Devido a sua relativa simplicidade técnica e reprodutibilidade, essa modalidade terapêutica pode ser utilizada como uma das opções de tratamento para pacientes portadores de telangiectasias (CEAP C1) e/ou varizes (CEAP C2) nos membros inferiores 3 ^,^ 4 . 

 A danificação endotelial pode ser alcançada tanto com a alteração do pH e da osmolaridade (que modificam a tensão superficial da membrana plasmática) quanto pela lesão direta do endotélio. Com esses propósitos, as soluções esclerosantes são agrupadas em três categorias: osmóticas, detergentes e irritantes químicos 1 . 

 As soluções osmóticas hipertônicas causam desidratação e desintegração endotelial e desnaturação da membrana celular. Atuam ainda na dispersão do fibrinogênio da camada íntima, depositando fibrina no interior e ao redor da parede venosa, provocando o seu colabamento e desaparecimento 5 ^-^ 9 . Objetivamente, sobrevém uma reação inflamatória que gradualmente evolui para fibrose. 

 Nesse sentido, a glicose hipertônica, introduzida por Kausch em 1917, é um dos agentes mais empregados no Brasil 10 , sendo rotineiramente usada em razão de sua eficácia, baixo custo e quase ausência de efeitos colaterais, como necrose ou reação alérgica. Todavia, pode requerer repetidas injeções no mesmo vaso em sessões subsequentes com intervalos variáveis 11 , o que, em tese, aumentaria o risco de complicações. 

 Por sua utilização ser incomum na Europa e nos Estados Unidos, a literatura concernente à glicose hipertônica é relativamente escassa 11 . Essa foi uma das razões que suscitaram descrever a ocorrência de uma complicação após seu emprego, tratada posteriormente com seu próprio uso. 

### Objetivo

 Relatar um caso de necrose cutânea após escleroterapia para telangiectasias nos membros inferiores utilizando glicose hipertônica (75%) e sua cicatrização empregando glicose 60% tópica. 

## DESCRIÇÃO DO CASO

 Paciente do sexo feminino, 49 anos, branca (fototipo III de Fitzpatrick), procurou atendimento médico em novembro de 2016 com queixa de varizes nos membros inferiores, então assintomáticas. Negava comorbidades ou alergias. Utilizava os medicamentos levonorgestrel 0,100 mg e etinilestradiol 0,020 mg. O exame físico demonstrou apenas telangiectasias em moderada quantidade (CEAP C1), predominantemente do tipo arboriforme. O exame arterial apresentou resultados normais. 

 Com finalidade essencialmente estética, em março de 2017 foi realizada a primeira escleroterapia com glicose 75% (previamente mantida à temperatura de 17 °C) utilizando agulha 0,40 x 13 mm (27G x ½”) e seringa de 3 mL (volume total = 2 mL). Cerca de 10 minutos após a injeção na região lateral da coxa direita, onde havia a presença de telangiectasias em maior concentração ( Figura 1 ), foi observada coloração de aspecto ocre que evoluiu com formação de bolhas e eritema fixo ( Figura 2 ), constatados no sétimo dia pós-escleroterapia (PE). 

**Figura 1:** Telangiectasias (tipo arborizada) e venulectasias na região lateral da coxa direita (CEAP 1).

**Figura 2:** Eritema e bolhas 7 dias após a injeção de glicose 75% para tratamento estético de telangiectasias CEAP 1.

 Apresentava ainda dor, edema (+ / +4) e empastamento (++ / +4) na panturrilha ipsilateral, iniciados simultaneamente. Foram realizados drenagem de trombos superficiais (mantendo as bolhas intactas) e exame com eco-Doppler colorido, devido à suspeita de trombose venosa profunda, que foi descartada. O uso da meia calça elástica (compressão 20-30 mmHg) foi recomendado após a escleroterapia inicial e proscrito após o sétimo dia PE, quando foram observados o edema e as lesões de pele. 

 No 14º dia PE, houve melhora da dor, do eritema e do edema, mas surgiram crostas ( Figura 3 ) em substituição às bolhas. A paciente foi orientada a realizar curativos diários utilizando óleo com ácidos graxos essenciais (AGEs). Devido à formação de necroses ( Figura 4 ), no 42º dia PE foi realizado desbridamento mecânico ( Figura 5 ), suspenso o uso de AGEs e iniciada a aplicação tópica diária de formulação contendo glicose 60% e vaselina 40% 12 ( Figura 6 ). 

**Figura 3:** Formação de crostas no 14º dia pós-escleroterapia com glicose hipertônica (75%).

**Figura 4:** Evolução de crostas para necrose no 33º dia pós-escleroterapia estética para telangiectasias no membros inferiores com glicose hipertônica (75%).

**Figura 5:** Pós-desbridamento mecânico de necrose cutânea causada por escleroterapia estética para telangiectasias nos membros inferiores com glicose hipertônica (75%).

**Figura 6:** Formulação tópica constituída de glicose 60% + vaselina 40%.

 Foi realizada uma segunda sessão de escleroterapia com glicose 75% no 49º dia PE, ainda com a presença de úlcera medindo 2,00 x 1,00 cm, evitando a injeção próxima à região. A paciente evoluiu com cicatrização da úlcera no 88º dia PE. Porém, restou hiperpigmentação ( Figura 7 ), sendo prescrita hidroquinona associada a ácido retinoico e hidrocortisona. 

**Figura 7:** Hiperpigmentação após cicatrização de úlcera cutânea utilizando formulação tópica contendo glicose 60%, causada por escleroterapia estética para telangiectasias nos membros inferiores com glicose hipertônica (75%).

 Após 6 meses de uso do despigmentante, a paciente apresentou discreta redução da pigmentação. Nesse mesmo período, foi realizada mais uma sessão de escleroterapia com glicose 75%, sem intercorrências. Decorrido 1 ano, houve clareamento moderado da pigmentação e da área da cicatriz ( Figura 8 ). Com relação às telangiectasias, o resultado foi relativamente satisfatório, com desaparecimento da maioria delas. 

**Figura 8:** Hiperpigmentação residual após necrose cutânea causada por escleroterapia estética para telangiectasias nos membros inferiores com glicose hipertônica (75%).

## DISCUSSÃO

 Os efeitos colaterais graves do tratamento escleroterápico são raros e frequentemente associados a um erro técnico ou à dosagem injetada. Suas complicações são predominantemente locais, como hiperpigmentação, *matting* e, mais raramente, necrose tissular e ulceração 1 , as quais são dolorosas e de lenta cicatrização. Algumas vezes, apresentam-se como uma situação de difícil resolução e de grande impacto psíquico, pois na quase totalidade dos casos a escleroterapia tem finalidade estética, porquanto as telangiectasias improvavelmente causariam sintomas. 

 O risco de reações indesejáveis relaciona-se diretamente ao tipo de esclerosante, ou seja, quanto maior a capacidade de lesar o endotélio, maior também a possibilidade de causar complicações. Nesse sentido, a glicose hipertônica tem-se demonstrado absolutamente segura 11 . Todavia, é descrita a possibilidade de formação de úlceras com a injeção de agentes osmóticos 13 . 

 Figueiredo e Figueiredo 10 analisaram, por meio de questionário, as condutas e técnicas de escleroterapia utilizadas pelos angiologistas e cirurgiões vasculares brasileiros. Nos resultados, 5,60% dos entrevistados afirmaram ter vivenciado a presença de úlceras acarretadas pela escleroterapia química convencional. Entretanto, não houve referência ao produto utilizado. 

 A necrose cutânea pode ocorrer com a injeção de qualquer agente esclerosante, mesmo nas condições técnicas ideais, e não representa, necessariamente, falha médica. Seu exato mecanismo de formação não é claramente conhecido. As causas sugeridas são: (1) extravasamento da solução para o espaço perivascular; (2) injeção em arteríola(s) dérmica(s) nutridora(s); (3) vasoespasmo reacional; (4) migração do esclerosante para o leito arterial (anastomoses arteriovenosas); (5) oclusão de *shunts* arteriovenosos; ou (6) pressão cutânea excessiva produzida por técnica compressiva externa inadequada 1 . 

 Miyake 14 associa o desenvolvimento da necrose cutânea ao refluxo venocapilar da solução esclerosante injetada (pressão excessiva), produzindo vasoconstrição e obstrução das microarteríolas regionais. Portanto, a necrose não resultaria da injeção intradérmica ou subcutânea inadvertida. Seria, na verdade, um fenômeno isquêmico, fato corroborado por outros autores 15 ^-^ 17 . A hipótese fundamentou-se em três pilares 17 :

1 – Presença de necrose cutânea mesmo quando não havia extravasamento da solução;
2 – O comportamento da necrose cutânea foi similar ao de úlceras isquêmicas causadas por oclusão arterial, tanto em termos de dor quanto de processo evolutivo;
3 – Quanto mais potente o esclerosante, maior a possibilidade de advir ulceração.

Estudos experimentais demonstram que a necrose cutânea relaciona-se diretamente com a pressão de injeção e inversamente com o diâmetro do vaso, isto é, quanto maior a pressão e menor o vaso, maior a possibilidade de sua ocorrência. De acordo com a lei de Poiseuille, a pressão diminui proporcionalmente ao aumento da viscosidade. Logo, o risco de necrose cutânea é menor quando se usa esclerosantes com alta viscosidade. Nesse sentido, os esclerosantes osmóticos são vantajosos em relação aos detergentes (menos viscosos) 1 ^,^ 17 . 

 Em contrapartida, Munavali e Weiss 18 sugeriram que a causa mais comum de necrose seria o extravasamento do esclerosante no território perivascular. O extravasamento causaria úlceras mais traumáticas do que isquêmicas, sendo mais comum com o uso de etanolamina, seguido por polidocanol, glicerina crômica e, por último, a glicose nas suas diversas concentrações hipertônicas, sendo esta considerada um dos agentes de maior segurança no que diz respeito a reações adversas, tanto locais quanto sistêmicas. 1 ^,^ 2 ^,^ 8 ^,^ 9 A glicose 75%, quando extravasada, causaria pequenas necroses de pele (1 a 2 mm), superficiais, que cicatrizariam em 1 a 2 semanas 1 ^,^ 17 . 

 Não obstante, fruto da observação prática, merece alusão que mesmo sem extravasamento e utilizando uma solução habitual, sem exercer pressão excessiva, existe a possibilidade do desenvolvimento de úlceras, ainda que o risco de necrose cutânea seja menor quando se usam esclerosantes com viscosidades maiores (glicose hipertônica). Uma explicação admissível seria o vasoespasmo reacional (reflexo venoarteriolar), provocando isquemia. Suspeitando-se dessa situação, o que nem sempre é simples, poder-se-ia tentar puncionar novamente a veia para lavar o território acometido com uma solução de soro fisiológico e lidocaína, devido ao seu forte efeito vasodilatador. 

 Por conseguinte, conjecturamos que a necrose de pele no caso exposto possivelmente deva ter sido causada por vasoespasmo reacional, especialmente como resultado do volume aplicado na região (próximo de 2,00 mL), já que não ocorreu alta pressão de injeção ou extravasamento da substância. Tampouco julgamos provável que haja sucedido: (1) injeção em arteríola dérmica (devido ao tipo azulado de telangiectasias); (2) oclusão de *shunts* arteriovenosos; ou (3) pressão excessiva causada pela meia elástica (20-30 mmHg) durante os primeiros 7 dias PE. Todavia, é importante realçar que a migração do esclerosante para o leito capilar ou arteriolar não poderia ser completamente desconsiderada, já que a avaliação da pressão exercida foi subjetiva. Além disso, pode ter ocorrido refluxo em decorrência da oclusão dos vasos tratados (em razão da quantidade injetada). 

 As úlceras superficiais, quando ocorrem, apresentam a possibilidade de serem tratadas com diversos produtos utilizados para regeneração tecidual, como cremes a base de vitamina A, D, aloe vera, óxido de zinco, entre outros. Úlceras mais profundas normalmente são acompanhadas de maior volume de tecido necrótico, necessitando desbridamento mecânico e/ou autolítico (fibrinolisina, colagenase, alginato de cálcio e sódio, papaína, etc.). 

 Até o momento, não há um consenso quanto ao curativo considerado ideal para o tratamento de úlceras de causa vascular, mormente em relação às causadas pela escleroterapia. Motivados pelos excelentes resultados de Franceschi et al. 12 , que aplicaram preparação farmacêutica contendo glicose com vaselina na terapia tópica de úlceras crônicas nos membros inferiores de distintas etiologias (trauma, isquemia, hipertensão venosa, etc.), e devido ao maior tamanho da necrose cutânea desenvolvida e da profundidade final da úlcera após o desbridamento mecânico, optamos por utilizar a mesma formulação, sem empregarmos qualquer tipo de droga sistêmica, inclusive antibióticos. Nessa combinação, a concentração de glicose é de 60%, enquanto encontra-se quase ausente no açúcar refinado e no mascavo, que contêm, respectivamente, 99,8% e 95% de sacarose 12 . Além disso, a mistura de vaselina e glicose não é alergênica e apresenta baixíssimo custo. 

 A eficiência anti-infecciosa e a aceleração da cicatrização propiciada pelo açúcar no tratamento de úlceras são conhecidas 19 ^-^ 21 . No relato apresentado, isso ocorreu em 46 dias. Entretanto, ao contrário do exposto por Franceschi et al. 12 , que trocavam o curativo a cada 6 ou 7 dias, optamos por substituí-lo a cada 24 horas estritamente devido à dificuldade de isolar a região da coxa durante o banho, o que manteria o local úmido. Apesar da atenuação moderada da hiperpigmentação pós-cicatricial, mantivemos o uso da hidroquinona associada ao ácido retinoico e à hidrocortisona, na expectativa de manter sua redução. 

 Em conclusão, a despeito da complicação narrada, a qual pode ter sido em decorrência de falha técnica prevenível, a glicose hipertônica ainda nos parece o esclerosante mais seguro em relação aos efeitos indesejáveis da escleroterapia. Embora possa parecer contraditório, a glicose tópica demonstrou ser eficiente na cicatrização da úlcera provocada pela sua própria injeção, com baixo custo e fácil aplicação.
